# Transcription factor CEBPB inhibits the proliferation of osteosarcoma by regulating downstream target gene CLEC5A

**DOI:** 10.1002/jcla.22985

**Published:** 2019-07-31

**Authors:** Jianhua Lu, Weikai Chen, Hao Liu, Huilin Yang, Tao Liu

**Affiliations:** ^1^ Department of Orthopaedic Surgery The First Affiliated Hospital of Soochow University Suzhou China

**Keywords:** C‐type lectin domain family 5, member A, enhancer‐binding protein beta, osteosarcoma, proliferation, transcription factor

## Abstract

**Objective:**

To screen the gene related to osteosarcoma (OS) metastasis and the molecular mechanism.

**Methods:**

GEO database and R2 chip analysis platform were used to screen genes related to OS metastasis. UCSC gene browser was used to find the transcription factor (TF) of CLEC5A. The mRNA level and protein expression of CLEC5A in OS tissues and normal tissues were determined by RT‐PCR and Western blotting, respectively. OS cell lines MG‐63 were transfected with CEBPB recombinant plasmid. After transfection, the expression of CLEC5A in MG‐63 cells was determined and the cell proliferation situation was determined by clone formation assay.

**Results:**

Three genes CLEC5A, ALOX5AP, and RNASE3 were obtained, and CLEC5A has the highest correlation with OS. CLEC5A has screened the gene related to OS metastasis, and CEBPB can be taken as TF regulating downstream gene CLEC5A. CEBPB can regulate the downstream CLEC5A as transcription factor. The relative mRNA level and protein expression of CLEC5A in OS tissues were significantly higher than those in normal tissues. CLEC5A can prevent OS metastasis. Transfection of CEBPB increased the expression of CLEC5A in MG‐63 cells and also inhibited the proliferation of OS.

**Conclusion:**

CEBPB can inhibit the proliferation of OS cells via regulating the expression of CLEC5A.

## 1 INTRODUCTION

Osteosarcoma (OS) is a primary malignant tumor of the skeleton characterized by the direct formation of immature bone or osteoid tissue in the tumor cells.^1^ More rarely, OS may arise in the soft tissue. About 75% of cases, patients with OS are between 15 and 25 years of age. Males are more frequently affected than females (ratio 1.5:1). OS observed in patients who usually develop secondary to Paget's disease, radiation, or dedifferentiated chondrosarcomas.^2^ Etiology of OS is unknown. The molecular level study on OS is still in its infancy, and the concrete mechanism remains to be further studied.

CLEC5A (C‐type lectin domain family 5, member A, also known as myeloid DAP12‐associating lectin (MDL‐1), contains a C‐type lectin‐like fold similar to the natural‐killer T‐cell C‐type lectin domains and associates with a 12‐kDa DNAX‐activating protein (DAP12) on myeloid cells.^3^ Studies have shown dengue virus (DV) can bind and activate CLEC5A and induce the phosphorylation of DAP12,^4^ which is responsible for CLEC5A/MDL‐1‐mediated signaling.^5^ CLEC5A regulates virus‐induced pro‐inflammatory cytokine release from macrophages. Blockade of CLEC5A can prevent autoimmune inflammation in collagen‐induced arthritis via down‐regulating osteoclast activation, suppressing cell infiltration of joints, and attenuating pro‐inflammatory cytokine release.^5^ In myeloid development, CLEC5A expression is associated with mature stages of myeloid differentiation.^4^ CLEC5A has an important function in innate immunity due to its role in macrophage and neutrophil differentiation as well as activation.^6^ The transcription factor (TF) CCAAT/enhancer‐binding protein beta (CEBPB) is important for maintaining the tumor‐initiating capacity and invasion ability.^7^ The expression of CEBPB mRNA and protein is markedly increased in several tumors.^8^

Through database screening, we found a gene CLEC5A related to OS, which was rarely reported and needed to further study. Moreover, we found the transcription factor CEBPB of CLEC5A, so we study whether the transcription factor CEBPB regulating CLEC5A has an effect on the proliferation of OS.

## 2 MATERIALS AND METHODS

### 2.1 Gene screening

Public dataset of gene expression profiles, GSE46448, was downloaded from GEO database (http://www.ncbi.nlm.nih.gov/geo). It includes 48 cases of Homo sapiens OS samples. Gene correlation analysis was performed by using R2 chip analysis platform (http://r2.amc.nl). The screening of differentially expressed genes was carried out by Significance Analysis of Microarrays (SAM) software algorithm.

### 2.2 Tissue samples

Microscopically confirmed tumor samples and paired adjacent normal tissues were obtained from 24 patients undergoing surgical resection of primary colorectal adenocarcinoma at Qilu Hospital, China. Following surgery, samples from tumor and adjacent normal tissues were frozen in liquid nitrogen and stored at −80°C for subsequent RNA extraction. Patient consent forms were obtained from all patients according to the institutional regulations.

### 2.3 Cell culture and transfection

The human osteosarcoma cell lines MG63 were maintained in DMEM nutrient mixture F‐12 plus 10% FBS. T‐47D cells were maintained in RPMI‐1640 medium plus 10% FBS, 5 μg/mL insulin, 10 mmol/L HEPES, and 1 mmol/L sodium pyruvate. Both breast cancer cell lines were maintained at 37°C and 5% CO_2_.

The CEBPB expression plasmid (SC119965) was synthesized by OriGene Tech, Inc. The Saos and MG63 cells were plated in 6‐well plates (1 × 10^5^ cells per well) and were transfected with CEBPB plasmid and mock plasmid vector using Lipofectamine 2000. Then, the cells were incubated in antibiotic‐free Opti‐MEM. After 48 hours of the cultivation, the cells were counted using TC10 Automated Cell Counter (Bio‐Rad).

### 2.4 RT‐PCR

Total RNA was isolated from tissue or cells with TRIzol kit. RT‐PCR was performed in a Mastercycler RT‐PCR detection system (Eppendorf). Relative expression level of genes was normalized against gene β‐actin by formula of 2^∆CT(β‐actin‐target)^. Gene level in experimental group was then normalized against the control group.

### 2.5 Western blotting

Tissues and cells were harvested and lysed in buffer (62.5 mmol/L Tris‐HCl, [pH 6.8], 10% glycerol, 2% SDS, 5% 2‐mercaptoethanol, and 0.5% bromphenol blue). Proteins were separated on 15% SDS‐polyacrylamide gels and electroblotted onto Immobilon‐P membranes (Millipore). Blots were incubated with a 1:1000 dilution of MAb 85.1 and detected by enhanced chemoluminescence according to the manufacturer's specifications (Amersham).

### 2.6 Clone formation assay

For the assay, 100 cells were plated in RPMI‐1640/10% FBS on six‐well plates per well and were cultured for 14 days. The number of the clones (≥50 cells) was assessed by counting under a microscope.

### 2.7 Statistical analysis

SPSS 22.0 software was used to perform *t* test and variance analysis. Significance was defined as *P* < 0.05.

## 3 RESULTS

### 3.1 Gene screening

**Figure 1:**
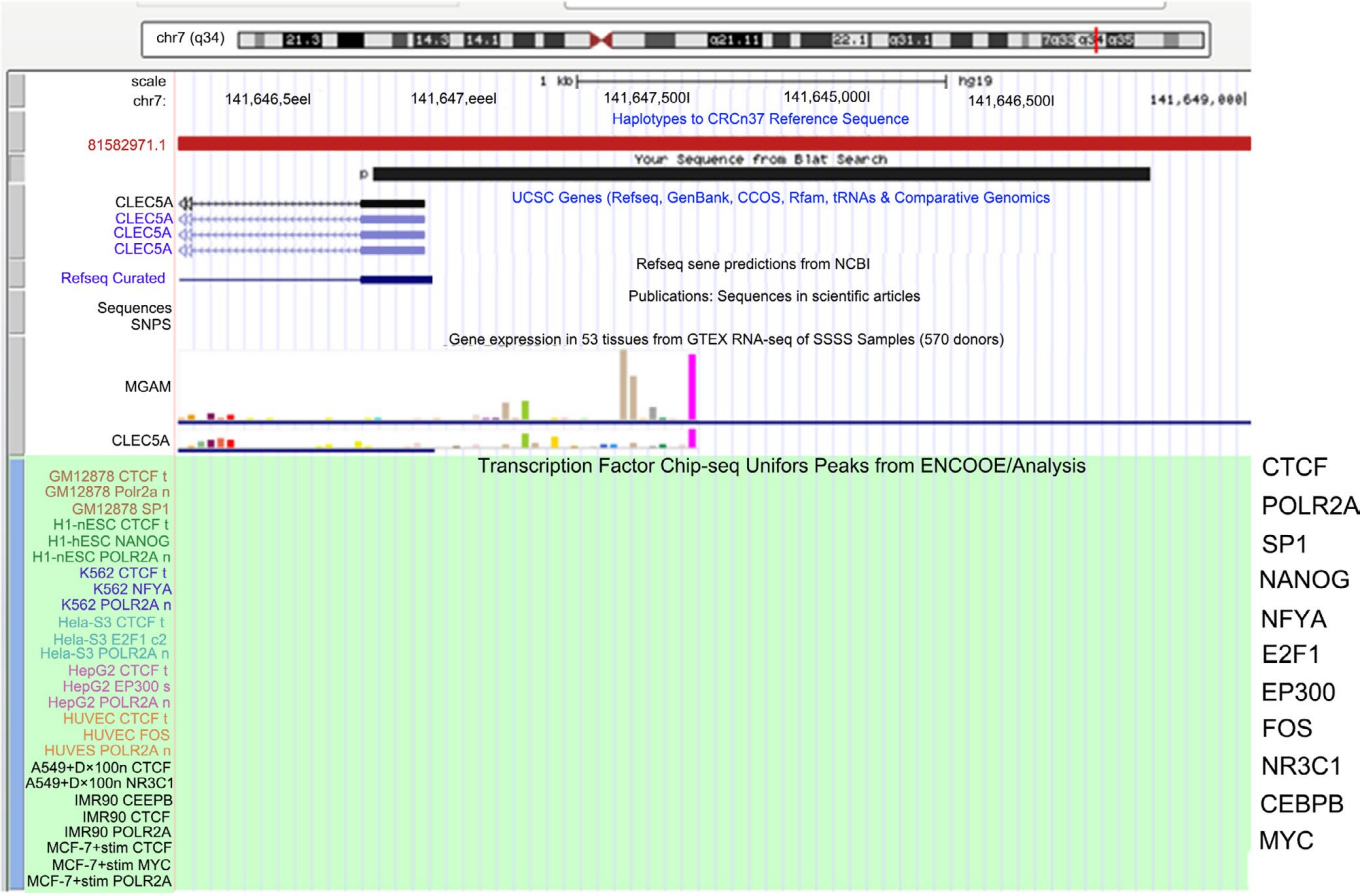
UCSC gene browser analysis to explore the transcription factors regulating CLEC5A

Genes associated with OS metastasis were screened using the R2 database, and we obtained three genes CLEC5A, ALOX5AP, and RNASE3. CLEC5A with the highest correlation was selected as the follow‐up research. UCSC gene browser was used to explore transcription factor regulating CLEC5A, and results are shown in Figure 1. Look up the correlation between transcription factors and CLEC5A in the database, and only CEBPB meets the requirements (*r* = .421, *P* = 1.68e‐03). In other words, CEBPB can regulate the downstream CLEC5A as transcription factor.

### 3.2 CLEC5A prevents OS metastasis

**Figure 2:**
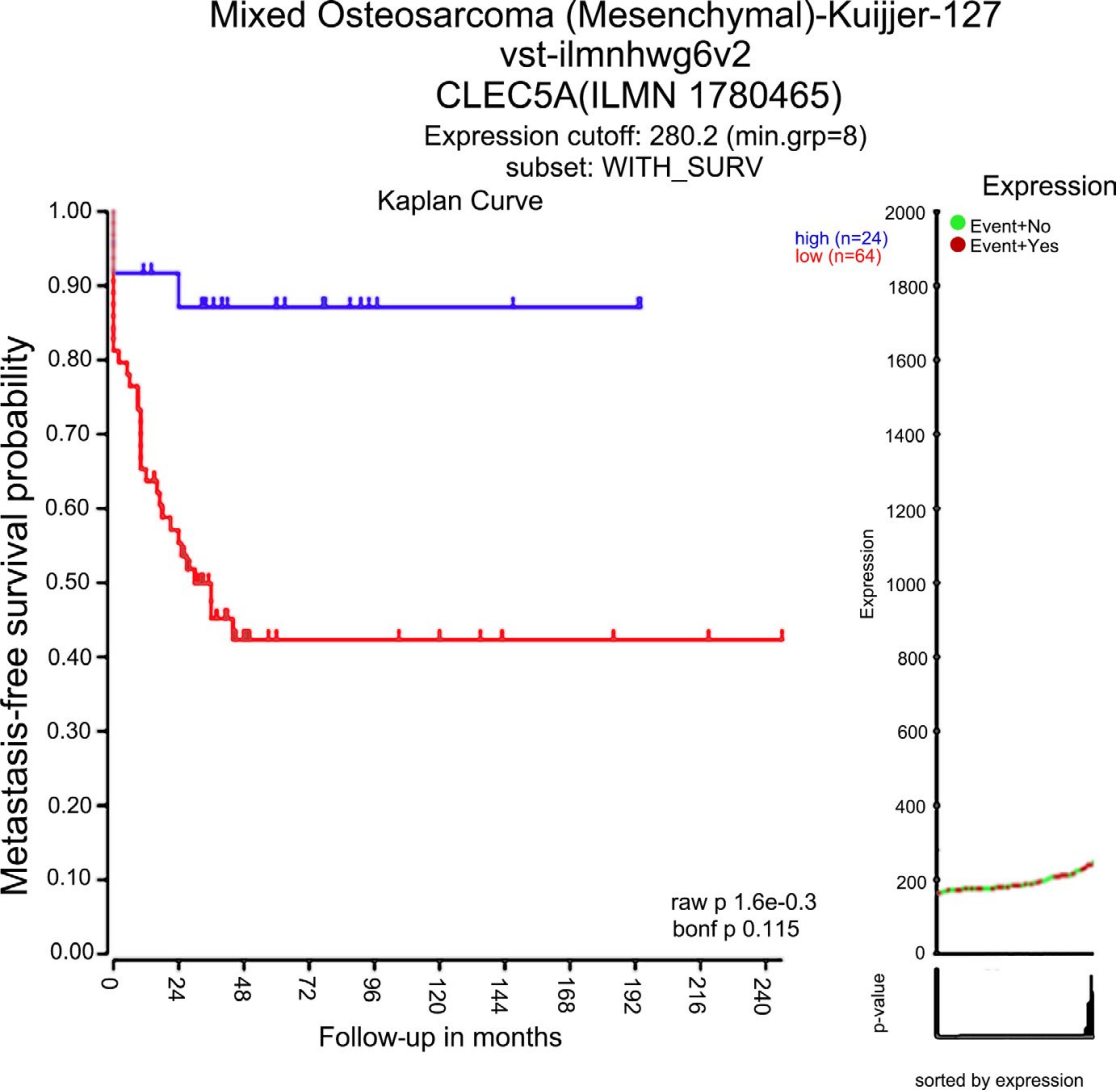
Effect of CLEC5A on the metastasis survival probability of osteosarcoma

Metastasis continues to be the leading cause of mortality for patients with cancer. Treatment‐refractory pulmonary metastasis continues to be the major complication of OS, reducing the 5‐year survival rate for these patients to 10%‐20%. We studied the effect of CLEC5A on OS metastasis, and results are shown in Figure 2. Results showed CLEC5A can prevent OS metastasis.

### 3.3 CLEC5A was down‐regulated in OS

**Figure 3:**
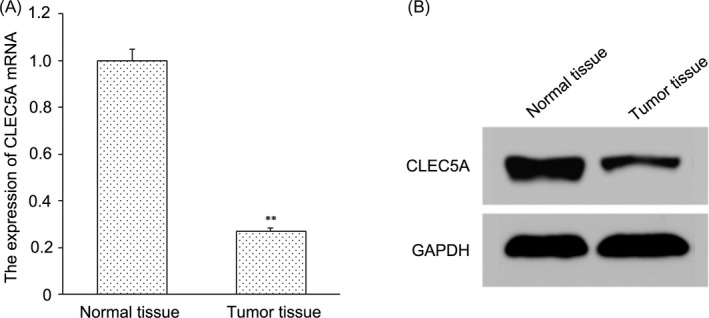
CLEC5A was down‐regulated in osteosarcoma. A, The relative mRNA level of CLEC5A in tumor tissues and the adjacent normal tissues by RT‐PCR. **P < 0.01 vs normal group*,* indicating the difference has statistical significance. B, The protein expression of CLEC5A in tumor tissue and the adjacent normal tissues by Western blotting assay

To explore the relationship between CLEC5A and OS, we determined the mRNA level and protein expression of CLEC5A in tumor tissues and the adjacent normal tissues by RT‐PCR and Western blotting, respectively. Results of RT‐PCR are shown in Figure 3A. The relative mRNA level of CLEC5A in tumor tissues was 0.27 ± 0.05, which was significantly lower than that in the normal tissues (*P* < 0.01). Similarly, the protein expression of CLEC5A in tumor tissues was also markedly decreased compared with the normal tissues (Figure 3B).

### 3.4 CEBPB is the transcription factor of CLEC5A

**Figure 4:**
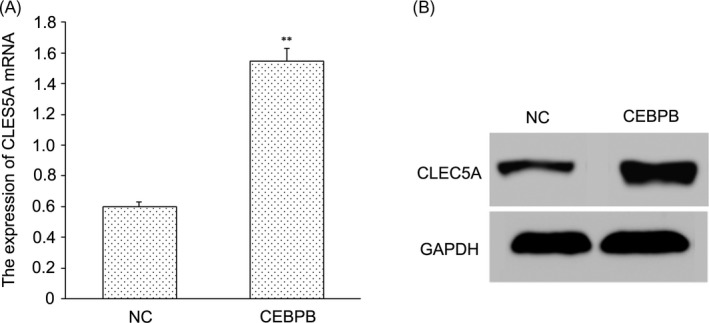
CEBPB is the transcription factor of CLEC5A. A, The relative mRNA level of CLEC5A in NC group and CEBPB group by RT‐PCR. **P < 0.01 vs NC group*,* indicating the difference has statistical significance. B, The protein expression of CLEC5A in NC group and CEBPB group by Western blotting assay

Transcription factor CEBPB recombinant plasmids were built, and recombinant plasmids were used to transfect OS cell line MG‐63. After transfection and culture, the relative mRNA level and protein expression of CLEC5A in the NC group and CEBPB group were determined by RT‐PCR and Western blotting, respectively. As results of RT‐PCR showed the relative mRNA level of CLEC5A in CEBPB group was observably increased compared with the NC group (Figure 4A), results of Western blotting were similar to those of RT‐PCR (Figure 4B).

### 3.5 CEBPB inhibits osteosarcoma cell proliferation

**Figure 5:**
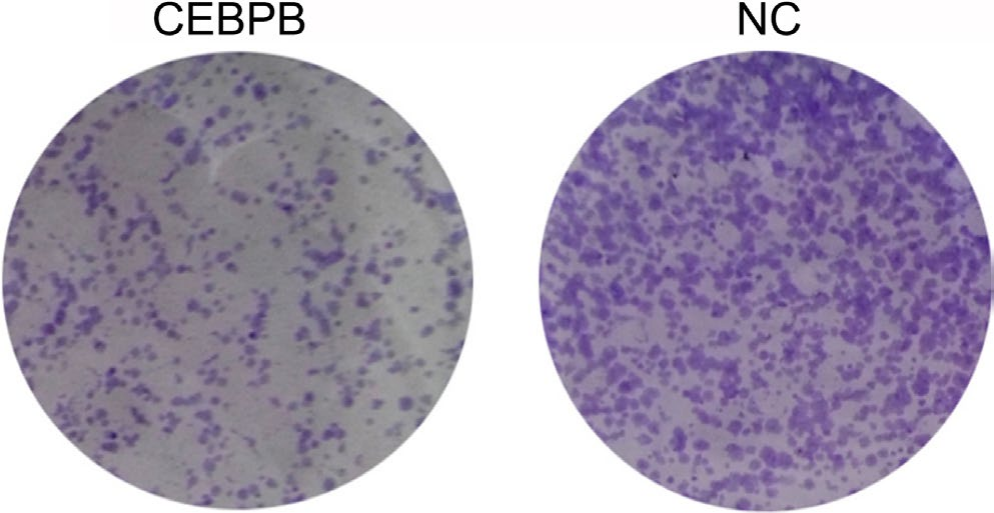
The clone formation situation of osteosarcoma cells MG‐63 in NC and CEBPB group was determined by clone formation assay

To explore the relationship between CEBPB and CLEC5A, and their role in the development of OS, we used clone formation assay to determine the cell proliferation situation after transfection with transcription factor CEBPB recombinant plasmids. Results showed the cell proliferation was markedly weakened after transfection with CEBPB recombinant plasmids (Figure 5), which indicated CEBPB can reduce OS cell proliferation by regulating CLEC5A.

## 4 DISCUSSION

OS derives from primitive bone‐forming mesenchymal cells and is the most common primary bone malignancy. Clinically, OS presents local pain and swelling with occasional joint dysfunction.^9^ Current optimal treatment for OS consists of multi‐agent chemotherapy and aggressive surgical resection of all sites of disease involvement. In addition to surgery, chemotherapy, and radiotherapy, the comprehensive treatment of OS also includes molecular targeted therapy, immunotherapy, gene therapy, embolization, radiofrequency ablation, and stem cell therapy.^10^ Pulmonary metastases are the main cause of death in patients with OS; however, the molecular mechanisms of metastasis are not well understood.^11^ Current treatments for OS are associated with significant morbidity, and a period of rehabilitation may be required following surgery for OS. Hence, there is a real need to optimize current treatment strategies and to develop novel approaches for treating OS.^12^ As our knowledge of the molecular pathogenesis of OS expands, potential therapeutic targets are being identified.^13^

Through analysis by GEO database and R2 chip analysis platform, we obtained three genes (CLEC5A, ALOX5AP, and RNASE3) associated with OS metastasis. CLEC5A with the highest correlation was selected for study. Then, we used UCSC gene browser to explore transcription factor regulating CLEC5A. Looking up the correlation between TF and CLEC5A in the database, only CEBPB meets the requirements. It indicates CEBPB can be taken as TF to regulate downstream CLEC5A. Moreover, our study showed CLEC5A plays a role in tumor inhibition in the metastasis of OS. CLEC5A is a C‐type lectin receptor implicated in the progression of multiple acute and chronic inflammatory diseases.^4^ It has now been identified that interacts with an envelope protein on the dengue virus to specifically induce the production of pro‐inflammatory cytokines by macrophages during infection.^14^ It is critical for dengue virus–induced osteoclast activation and bone homeostasis. CCAAT/enhancer‐binding protein beta (CEBPB) is a key factor of Runx2 expression.^15^ It is necessary for M2 macrophage–mediated regeneration after muscle injury. In humans, CEBPB expression in blood was strongly associated with muscle strength.^16^ CEBPB is important for maintaining the tumor‐initiating capacity and invasion ability.^7^ We forecast whether CEBPB can inhibit the growth of osteosarcoma by promoting the expression of downstream CLEC5A. We determined the expression of CLEC5A in OS tumor tissues and the adjacent normal tissues by RT‐PCR and Western blotting. Results showed the expression of CLEC5A was markedly decreased in tumor tissues compared with the normal tissues. We obtained CLEC5A was up‐regulated stably in OS. Then, we built transcription factor CEBPB recombinant plasmid and transfected it to osteosarcoma cell line MG‐63. After transfection with CEBPB recombinant plasmid, we found the expression of CLEC5A was significantly reduced compared with the control group. Moreover, transfection with CEBPB recombinant plasmid reduced the proliferation of OS cells.

In conclusion, CLEC5A was down‐regulated in osteosarcoma. CEBPB can inhibit the proliferation of osteosarcoma via regulating the expression of CLEC5A.

## CONFLICT OF INTEREST

All of the authors have no conflict of interest in this research.

## AUTHORS CONTRIBUTION

Each author has made an important scientific contribution to the study and has assisted with the drafting or revising of the article.

## ETHICS, CONSENT, AND PERMISSIONS

Ethical approval was given by The First Affiliated Hospital of Soochow University.

## CONSENT TO PUBLISH

All of the authors have consented to publish this research.
